# Di-μ-thio­cyanato-κ^4^ *N*:*N*-bis­({5-meth­oxy-2-[3-(methyl­amino)propyl­imino­meth­yl]phenolato-κ^3^ *O* ^1^,*N*,*N*′}copper(II))

**DOI:** 10.1107/S1600536810015564

**Published:** 2010-04-30

**Authors:** Nong Wang, Rui Xue, Bo Li, Yu-Ping Yang, Min Cao

**Affiliations:** a School of Chemical and Biological Engineering, Lanzhou Jiaotong University, Lanzhou 730070, People’s Republic of China

## Abstract

The title thio­cyanate-bridged dinuclear copper(II) complex, [Cu_2_(C_12_H_17_N_2_O_2_)_2_(NCS)_2_], possesses crystallographic inversion symmetry. Each Cu^II^ atom is five-coordinated by one imine N, one amine N and one phenolate O atom of the Schiff base ligand, and by two N atoms from two bridging thio­cyanate ligands, forming a square-pyramidal geometry. Beside the two thio­cyanate bridges, there are two intra­molecular N—H⋯O hydrogen bonds, which further link the two Cu(C_12_H_17_N_2_O_2_)(NCS) units. The Cu⋯Cu separation is 3.261 (2) Å. Parts of the methylaminopropylimino segment are disordered over two sites with occupancies of 0.669(9) and 0.331(9).

## Related literature

For general background to copper complexes, see: Reddy *et al.* (2000 ▶); Ray *et al.* (2003 ▶); Arnold *et al.* (2003 ▶); Raptopoulou *et al.* (1998 ▶). For our previous reports of copper(II) complexes, see: Wang & Li (2005 ▶); Wang *et al.* (2006 ▶). For related structures, see: Elmali *et al.* (2000 ▶); You & Zhu (2005 ▶); Liu *et al.* (2004 ▶); Datta *et al.* (2008 ▶); Habibi *et al.* (2007 ▶).
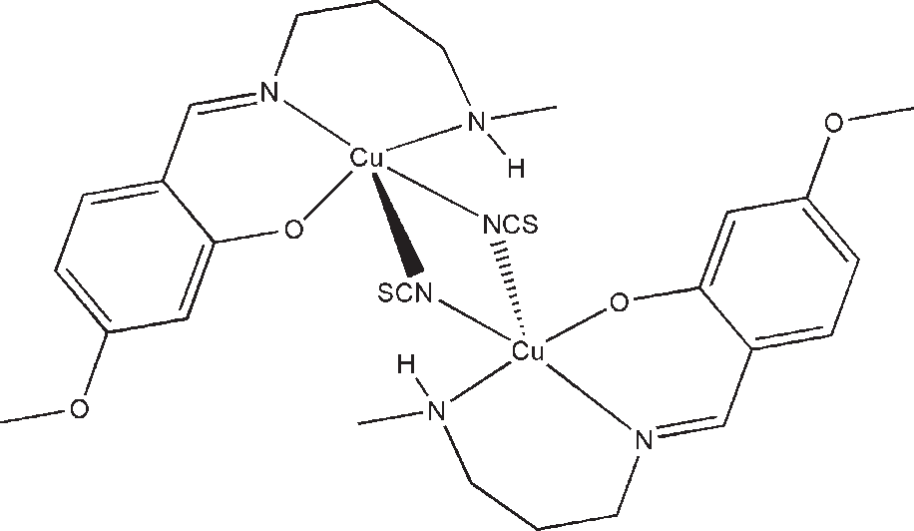


## Experimental

### 

#### Crystal data

[Cu_2_(C_12_H_17_N_2_O_2_)_2_(NCS)_2_]
*M* *_r_* = 685.79
Monoclinic, 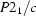

*a* = 11.8003 (18) Å
*b* = 15.373 (2) Å
*c* = 8.6740 (13) Å
β = 108.972 (7)°
*V* = 1488.0 (4) Å^3^
*Z* = 2
Mo *K*α radiation
μ = 1.61 mm^−1^
*T* = 298 K
0.20 × 0.20 × 0.18 mm

#### Data collection

Bruker SMART CCD area-detector diffractometer
Absorption correction: multi-scan (*SADABS*; Sheldrick, 1996 ▶) *T*_min_ = 0.739, *T*_max_ = 0.760
9297 measured reflections
3544 independent reflections
2496 reflections with *I* > 2σ(*I*)
*R*_int_ = 0.030

#### Refinement

*R*[*F* ^2^ > 2σ(*F* ^2^)] = 0.040
*wR*(*F* ^2^) = 0.108
*S* = 1.05
3544 reflections
211 parameters
50 restraints
H-atom parameters constrained
Δρ_max_ = 0.48 e Å^−3^
Δρ_min_ = −0.41 e Å^−3^

### 

Data collection: *SMART* (Bruker, 1998 ▶); cell refinement: *SAINT* (Bruker, 1998 ▶); data reduction: *SAINT*; program(s) used to solve structure: *SHELXS97* (Sheldrick, 2008 ▶); program(s) used to refine structure: *SHELXL97* (Sheldrick, 2008 ▶); molecular graphics: *SHELXTL* (Sheldrick, 2008 ▶); software used to prepare material for publication: *SHELXTL*.

## Supplementary Material

Crystal structure: contains datablocks global, I. DOI: 10.1107/S1600536810015564/ci5079sup1.cif

Structure factors: contains datablocks I. DOI: 10.1107/S1600536810015564/ci5079Isup2.hkl

Additional supplementary materials:  crystallographic information; 3D view; checkCIF report

## Figures and Tables

**Table 1:** Selected bond lengths (Å)

Cu1—O1	1.910 (2)
Cu1—N1	1.953 (2)
Cu1—N2	1.997 (3)
Cu1—N3	1.998 (3)
Cu1—N3^i^	2.598 (4)

Symmetry code: (i) 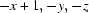.

**Table 2:** Hydrogen-bond geometry (Å, °)

*D*—H⋯*A*	*D*—H	H⋯*A*	*D*⋯*A*	*D*—H⋯*A*
N2—H2*A*⋯O1^i^	0.91	2.14	2.999 (3)	157

Symmetry code: (i) 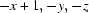.

**Fig. 1.**
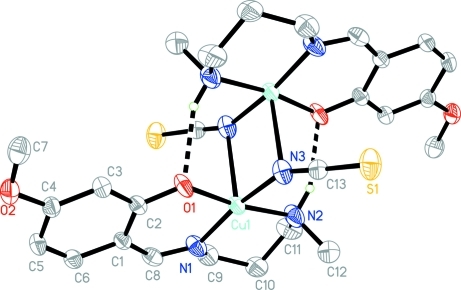
The molecular structure of the title compound, showing 30% displacement ellipsoids (arbitrary spheres for the H atoms). Unlabelled atoms are at the symmetry position (1 - x, - y, - z). Only the major disorder component is shown.

**Fig. 2.**
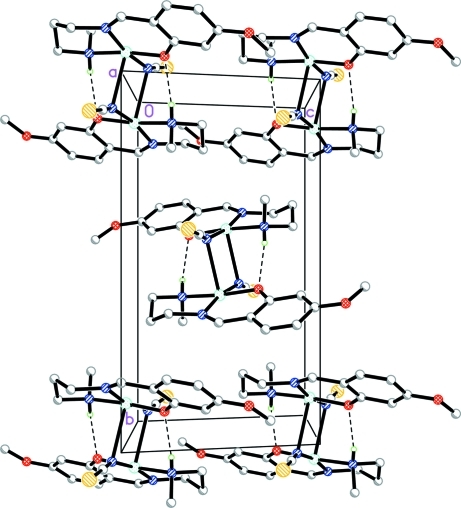
The crystal packing of the title compound. Intramolecular N—H···O hydrogen bonds are shown as dashed lines.

## supplementary crystallographic information

### Comment

An extensive effort has been made to prepare and characterize a variety of coordination complexes in an attempt to model the physical and chemical behaviour of copper-containing enzymes (Reddy *et al.*, 2000). The peculiarity of copper lies in its ability to form complexes with coordination numbers of four, five, and six (Ray *et al.* 2003; Arnold *et al.*, 2003; Raptopoulou *et al.*, 1998). As a continuation of our own work in this area (Wang & Li, 2005; Wang *et al.*, 2006), the title compound, a new copper(II) complex, is reported here.

The title compound is a thiocyanate-bridged dinuclear copper(II) complex (Fig. 1), with a Cu···Cu separation of 3.2608 (7) Å. The complex possesses a crystallographic inversion centre symmetry. Each Cu^II^ atom is five-coordinated by one imine N, one amine N, and one phenolate O atom of the Schiff base ligand, and by two N atoms from two thiocyanate ligands, forming a square-pyramidal geometry. The bond lengths and angles (Table 1) are typical and comparable with those in other copper(II) complexes with Schiff bases and thiocyanate ligands (Elmali *et al.*, 2000; You & Zhu, 2005; Liu *et al.*, 2004; Datta *et al.*, 2008; Habibi *et al.*, 2007). Beside the two thiocyanate bridges, there exist two N—H···O hydrogen bonds (Table 2) in the complex, which further link the two [Cu(C_12_H_17_N_2_O_2_)(NCS)] units together (Fig. 2).

### Experimental

4-Methoxysalicylaldehyde (0.1 mmol, 15.2 mg), *N*-methylpropane-1,3-diamine (0.1 mmol, 8.8 mg), NH_4_NCS (0.1 mmol, 7.6 mg) and Cu(CH_3_COO)_2_.H_2_O (0.1 mmol, 19.9 mg) were dissolved in methanol (20 ml). The mixture was stirred at room temperature for 1 h to give a blue solution. The resulting solution was allowed to stand in air for a few days, and blue block-shaped crystals were formed.

### Refinement

Atoms C9, C10 and C11 of the methylaminopropylimino segment are disordered over two sites with occupancies of 0.669 (9) and 0.331 (9). The N—C and also the C—C distances involving the disordered atoms were restrained to be equal. The U^ij^ parameters of the disordered atoms, and atom C12 were restrained to an approximate isotropic behaviour. H atoms were placed in idealized positions and constrained to ride on their parent atoms, with C—H distances in the range 0.93-0.97 Å, N—H distances in the range 0.90-0.91 Å and with U_iso_(H) = 1.2U_eq_(C,N) and 1.5U_eq_(methyl C).

### Crystal data

**Table:**

[Cu_2_(C_12_H_17_N_2_O_2_)_2_(NCS)_2_]	*F*(000) = 708
*M**_r_* = 685.79	*D*_x_ = 1.531 Mg m^−^^3^
Monoclinic, *P*2_1_/*c*	Mo *K*α radiation, λ = 0.71073 Å
Hall symbol: -P 2ybc	Cell parameters from 2304 reflections
*a* = 11.8003 (18) Å	θ = 2.5–25.1°
*b* = 15.373 (2) Å	µ = 1.61 mm^−^^1^
*c* = 8.6740 (13) Å	*T* = 298 K
β = 108.972 (7)°	Block, blue
*V* = 1488.0 (4) Å^3^	0.20 × 0.20 × 0.18 mm
*Z* = 2	

### Data collection

**Table:**

Bruker SMART CCD area-detector diffractometer	3544 independent reflections
Radiation source: fine-focus sealed tube	2496 reflections with *I* > 2σ(*I*)
graphite	*R*_int_ = 0.030
ω scans	θ_max_ = 28.5°, θ_min_ = 1.8°
Absorption correction: multi-scan (*SADABS*; Sheldrick, 1996)	*h* = −15→15
*T*_min_ = 0.739, *T*_max_ = 0.760	*k* = −20→19
9297 measured reflections	*l* = −11→10

### Refinement

**Table:**

Refinement on *F*^2^	Primary atom site location: structure-invariant direct methods
Least-squares matrix: full	Secondary atom site location: difference Fourier map
*R*[*F*^2^ > 2σ(*F*^2^)] = 0.040	Hydrogen site location: inferred from neighbouring sites
*wR*(*F*^2^) = 0.108	H-atom parameters constrained
*S* = 1.05	*w* = 1/[σ^2^(*F*_o_^2^) + (0.0508*P*)^2^ + 0.2175*P*] where *P* = (*F*_o_^2^ + 2*F*_c_^2^)/3
3544 reflections	(Δ/σ)_max_ = 0.001
211 parameters	Δρ_max_ = 0.48 e Å^−^^3^
50 restraints	Δρ_min_ = −0.41 e Å^−^^3^

### Special details

**Table:**

Geometry. All esds (except the esd in the dihedral angle between two l.s. planes) are estimated using the full covariance matrix. The cell esds are taken into account individually in the estimation of esds in distances, angles and torsion angles; correlations between esds in cell parameters are only used when they are defined by crystal symmetry. An approximate (isotropic) treatment of cell esds is used for estimating esds involving l.s. planes.
Refinement. Refinement of *F*^2^ against ALL reflections. The weighted *R*-factor wR and goodness of fit *S* are based on *F*^2^, conventional *R*-factors *R* are based on *F*, with *F* set to zero for negative *F*^2^. The threshold expression of *F*^2^ > σ(*F*^2^) is used only for calculating *R*-factors(gt) etc. and is not relevant to the choice of reflections for refinement. *R*-factors based on *F*^2^ are statistically about twice as large as those based on *F*, and *R*- factors based on ALL data will be even larger.

### Fractional atomic coordinates and isotropic or equivalent isotropic displacement parameters (Å^2^)

**Table:**

	*x*	*y*	*z*	*U*_iso_*/*U*_eq_	Occ. (<1)
Cu1	0.44981 (3)	0.09693 (2)	0.01842 (4)	0.04380 (14)	
S1	0.77288 (9)	0.09042 (7)	−0.17937 (13)	0.0751 (3)	
O1	0.33324 (18)	0.07895 (14)	−0.1912 (2)	0.0557 (6)	
O2	−0.03088 (19)	0.11364 (15)	−0.6308 (3)	0.0612 (6)	
N1	0.3347 (2)	0.14948 (17)	0.1093 (3)	0.0578 (7)	
N2	0.5877 (3)	0.1033 (2)	0.2257 (4)	0.0775 (10)	
H2A	0.6321	0.0548	0.2262	0.093*	0.669 (9)
H2B	0.6324	0.0572	0.2174	0.093*	0.331 (9)
N3	0.5707 (2)	0.06534 (19)	−0.0884 (3)	0.0608 (7)	
C1	0.1697 (2)	0.15468 (16)	−0.1467 (3)	0.0412 (6)	
C2	0.2230 (2)	0.10933 (17)	−0.2462 (4)	0.0433 (7)	
C3	0.1564 (3)	0.09443 (17)	−0.4110 (4)	0.0447 (7)	
H3	0.1906	0.0638	−0.4774	0.054*	
C4	0.0408 (2)	0.12515 (19)	−0.4741 (4)	0.0458 (7)	
C5	−0.0121 (3)	0.1713 (2)	−0.3762 (4)	0.0558 (8)	
H5	−0.0897	0.1925	−0.4201	0.067*	
C6	0.0507 (3)	0.18466 (18)	−0.2176 (4)	0.0516 (7)	
H6	0.0145	0.2146	−0.1528	0.062*	
C7	0.0149 (3)	0.0685 (2)	−0.7408 (4)	0.0664 (9)	
H7A	0.0827	0.0993	−0.7513	0.080*	
H7B	−0.0461	0.0646	−0.8454	0.080*	
H7C	0.0391	0.0110	−0.6998	0.080*	
C8	0.2262 (3)	0.16936 (18)	0.0221 (4)	0.0516 (7)	
H8	0.1799	0.1965	0.0768	0.062*	
C9	0.3506 (5)	0.1469 (5)	0.2885 (6)	0.0633 (17)	0.669 (9)
H9A	0.3322	0.0889	0.3178	0.076*	0.669 (9)
H9B	0.2951	0.1872	0.3117	0.076*	0.669 (9)
C10	0.4770 (5)	0.1705 (5)	0.3900 (8)	0.068 (2)	0.669 (9)
H10A	0.4972	0.2259	0.3522	0.082*	0.669 (9)
H10B	0.4798	0.1780	0.5022	0.082*	0.669 (9)
C11	0.5706 (8)	0.1048 (6)	0.3857 (7)	0.080 (3)	0.669 (9)
H11A	0.6459	0.1191	0.4689	0.096*	0.669 (9)
H11B	0.5462	0.0475	0.4098	0.096*	0.669 (9)
C9A	0.3884 (12)	0.1980 (8)	0.2722 (11)	0.068 (4)	0.331 (9)
H9AA	0.4489	0.2391	0.2655	0.082*	0.331 (9)
H9AB	0.3265	0.2293	0.3004	0.082*	0.331 (9)
C10A	0.4432 (15)	0.1287 (10)	0.397 (2)	0.086 (5)	0.331 (9)
H10C	0.3808	0.0888	0.4019	0.103*	0.331 (9)
H10D	0.4758	0.1559	0.5033	0.103*	0.331 (9)
C11A	0.5414 (14)	0.0777 (10)	0.3617 (15)	0.085 (6)	0.331 (9)
H11C	0.6096	0.0773	0.4613	0.102*	0.331 (9)
H11D	0.5137	0.0181	0.3414	0.102*	0.331 (9)
C12	0.6695 (4)	0.1786 (3)	0.2263 (7)	0.1234 (19)	
H12A	0.6282	0.2323	0.2276	0.148*	
H12B	0.6930	0.1762	0.1303	0.148*	
H12C	0.7394	0.1753	0.3214	0.148*	
C13	0.6548 (3)	0.07722 (19)	−0.1263 (4)	0.0500 (7)	

### Atomic displacement parameters (Å^2^)

**Table:**

	*U*^11^	*U*^22^	*U*^33^	*U*^12^	*U*^13^	*U*^23^
Cu1	0.0406 (2)	0.0484 (2)	0.0408 (2)	0.00958 (15)	0.01100 (16)	−0.00055 (14)
S1	0.0543 (5)	0.1109 (8)	0.0669 (6)	−0.0065 (5)	0.0290 (5)	0.0033 (5)
O1	0.0440 (12)	0.0789 (15)	0.0409 (11)	0.0279 (10)	0.0095 (9)	−0.0046 (10)
O2	0.0424 (12)	0.0714 (15)	0.0598 (15)	0.0034 (10)	0.0026 (11)	−0.0030 (11)
N1	0.0597 (16)	0.0727 (18)	0.0399 (14)	0.0239 (14)	0.0149 (13)	−0.0057 (12)
N2	0.0566 (18)	0.093 (2)	0.063 (2)	0.0254 (16)	−0.0074 (15)	−0.0285 (16)
N3	0.0474 (15)	0.0776 (18)	0.0592 (17)	0.0080 (14)	0.0195 (14)	0.0049 (14)
C1	0.0389 (14)	0.0394 (14)	0.0484 (16)	0.0053 (12)	0.0185 (13)	0.0028 (12)
C2	0.0404 (15)	0.0425 (16)	0.0472 (17)	0.0080 (12)	0.0146 (13)	0.0073 (12)
C3	0.0411 (15)	0.0495 (16)	0.0435 (16)	0.0043 (12)	0.0140 (13)	0.0037 (12)
C4	0.0387 (16)	0.0446 (15)	0.0500 (17)	0.0000 (12)	0.0088 (14)	0.0074 (13)
C5	0.0328 (15)	0.0577 (19)	0.073 (2)	0.0075 (13)	0.0113 (16)	0.0021 (16)
C6	0.0410 (16)	0.0481 (17)	0.070 (2)	0.0043 (13)	0.0239 (16)	−0.0047 (14)
C7	0.061 (2)	0.079 (2)	0.0491 (19)	−0.0022 (18)	0.0044 (17)	0.0007 (17)
C8	0.0577 (19)	0.0498 (17)	0.0539 (18)	0.0126 (14)	0.0271 (16)	0.0011 (14)
C9	0.072 (4)	0.075 (4)	0.044 (3)	0.013 (3)	0.021 (3)	−0.004 (3)
C10	0.080 (4)	0.073 (4)	0.042 (3)	0.010 (3)	0.007 (3)	−0.021 (3)
C11	0.104 (5)	0.086 (5)	0.030 (3)	0.029 (4)	−0.007 (3)	−0.017 (3)
C9A	0.070 (7)	0.080 (7)	0.053 (6)	0.028 (6)	0.018 (5)	−0.008 (5)
C10A	0.089 (9)	0.106 (9)	0.062 (7)	0.005 (7)	0.024 (7)	0.018 (7)
C11A	0.100 (9)	0.085 (8)	0.038 (7)	0.041 (7)	−0.022 (6)	−0.032 (6)
C12	0.066 (3)	0.138 (4)	0.148 (4)	−0.020 (3)	0.010 (3)	−0.070 (3)
C13	0.0459 (17)	0.0568 (18)	0.0452 (17)	0.0038 (14)	0.0118 (15)	0.0024 (13)

### Geometric parameters (Å, °)

**Table:**

Cu1—O1	1.910 (2)	C5—H5	0.93
Cu1—N1	1.953 (2)	C6—H6	0.93
Cu1—N2	1.997 (3)	C7—H7A	0.96
Cu1—N3	1.998 (3)	C7—H7B	0.96
Cu1—N3^i^	2.598 (4)	C7—H7C	0.96
S1—C13	1.616 (3)	C8—H8	0.93
O1—C2	1.317 (3)	C9—C10	1.509 (6)
O2—C4	1.359 (3)	C9—H9A	0.97
O2—C7	1.421 (4)	C9—H9B	0.97
N1—C8	1.294 (4)	C10—C11	1.506 (6)
N1—C9	1.504 (5)	C10—H10A	0.97
N1—C9A	1.540 (8)	C10—H10B	0.97
N2—C11	1.467 (6)	C11—H11A	0.97
N2—C12	1.505 (5)	C11—H11B	0.97
N2—C11A	1.506 (9)	C9A—C10A	1.507 (8)
N2—H2A	0.91	C9A—H9AA	0.97
N2—H2B	0.90	C9A—H9AB	0.97
N3—C13	1.157 (4)	C10A—C11A	1.510 (8)
C1—C2	1.407 (4)	C10A—H10C	0.97
C1—C6	1.415 (4)	C10A—H10D	0.97
C1—C8	1.416 (4)	C11A—H11C	0.97
C2—C3	1.408 (4)	C11A—H11D	0.97
C3—C4	1.378 (4)	C12—H12A	0.96
C3—H3	0.93	C12—H12B	0.96
C4—C5	1.399 (4)	C12—H12C	0.96
C5—C6	1.350 (4)		
			
O1—Cu1—N1	93.67 (10)	H7A—C7—H7B	109.5
O1—Cu1—N2	171.12 (10)	O2—C7—H7C	109.5
N1—Cu1—N2	94.96 (12)	H7A—C7—H7C	109.5
O1—Cu1—N3	85.70 (10)	H7B—C7—H7C	109.5
N1—Cu1—N3	169.45 (12)	N1—C8—C1	127.6 (3)
N2—Cu1—N3	86.20 (12)	N1—C8—H8	116.2
N3^i^—Cu1—N1	99.91 (15)	C1—C8—H8	116.2
N3^i^—Cu1—N2	87.06 (15)	N1—C9—C10	111.3 (5)
N3^i^—Cu1—N3	90.57 (15)	N1—C9—H9A	109.4
N3^i^—Cu1—O1	89.50 (15)	C10—C9—H9A	109.4
C2—O1—Cu1	127.91 (18)	N1—C9—H9B	109.4
C4—O2—C7	119.1 (2)	C10—C9—H9B	109.4
C8—N1—C9	112.2 (3)	H9A—C9—H9B	108.0
C8—N1—C9A	117.1 (5)	C11—C10—C9	114.7 (6)
C8—N1—Cu1	123.2 (2)	C11—C10—H10A	108.6
C9—N1—Cu1	122.4 (3)	C9—C10—H10A	108.6
C9A—N1—Cu1	116.0 (5)	C11—C10—H10B	108.6
C11—N2—C12	105.7 (5)	C9—C10—H10B	108.6
C12—N2—C11A	126.4 (7)	H10A—C10—H10B	107.6
C11—N2—Cu1	122.0 (4)	N2—C11—C10	111.2 (5)
C12—N2—Cu1	112.0 (3)	N2—C11—H11A	109.4
C11A—N2—Cu1	107.2 (7)	C10—C11—H11A	109.4
C11—N2—H2A	105.3	N2—C11—H11B	109.4
C12—N2—H2A	105.3	C10—C11—H11B	109.4
C11A—N2—H2A	97.8	H11A—C11—H11B	108.0
Cu1—N2—H2A	105.3	C10A—C9A—N1	105.7 (11)
C11—N2—H2B	110.6	C10A—C9A—H9AA	110.6
C12—N2—H2B	102.4	N1—C9A—H9AA	110.6
C11A—N2—H2B	103.1	C10A—C9A—H9AB	110.6
Cu1—N2—H2B	102.5	N1—C9A—H9AB	110.6
C13—N3—Cu1	154.3 (3)	H9AA—C9A—H9AB	108.7
C2—C1—C6	118.3 (3)	C9A—C10A—C11A	113.5 (12)
C2—C1—C8	124.0 (3)	C9A—C10A—H10C	108.9
C6—C1—C8	117.7 (3)	C11A—C10A—H10C	108.9
O1—C2—C1	122.6 (3)	C9A—C10A—H10D	108.9
O1—C2—C3	118.0 (3)	C11A—C10A—H10D	108.9
C1—C2—C3	119.3 (3)	H10C—C10A—H10D	107.7
C4—C3—C2	120.1 (3)	N2—C11A—C10A	121.4 (11)
C4—C3—H3	120.0	N2—C11A—H11C	107.0
C2—C3—H3	120.0	C10A—C11A—H11C	107.0
O2—C4—C3	124.5 (3)	N2—C11A—H11D	107.0
O2—C4—C5	114.7 (2)	C10A—C11A—H11D	107.0
C3—C4—C5	120.8 (3)	H11C—C11A—H11D	106.7
C6—C5—C4	119.4 (3)	N2—C12—H12A	109.5
C6—C5—H5	120.3	N2—C12—H12B	109.5
C4—C5—H5	120.3	H12A—C12—H12B	109.5
C5—C6—C1	122.1 (3)	N2—C12—H12C	109.5
C5—C6—H6	118.9	H12A—C12—H12C	109.5
C1—C6—H6	118.9	H12B—C12—H12C	109.5
O2—C7—H7A	109.5	N3—C13—S1	178.1 (3)
O2—C7—H7B	109.5		
			
N1—Cu1—O1—C2	10.5 (3)	C7—O2—C4—C5	179.3 (3)
N3—Cu1—O1—C2	−159.0 (3)	C2—C3—C4—O2	−179.5 (3)
O1—Cu1—N1—C8	−8.2 (3)	C2—C3—C4—C5	0.0 (4)
N2—Cu1—N1—C8	173.9 (3)	O2—C4—C5—C6	178.6 (3)
N3—Cu1—N1—C8	78.0 (7)	C3—C4—C5—C6	−0.9 (4)
O1—Cu1—N1—C9	153.7 (4)	C4—C5—C6—C1	1.0 (5)
N2—Cu1—N1—C9	−24.2 (4)	C2—C1—C6—C5	−0.2 (4)
N3—Cu1—N1—C9	−120.1 (6)	C8—C1—C6—C5	−177.8 (3)
O1—Cu1—N1—C9A	−165.8 (6)	C9—N1—C8—C1	−160.8 (4)
N2—Cu1—N1—C9A	16.3 (6)	C9A—N1—C8—C1	160.1 (7)
N3—Cu1—N1—C9A	−79.5 (8)	Cu1—N1—C8—C1	2.8 (5)
N1—Cu1—N2—C11	25.6 (5)	C2—C1—C8—N1	4.5 (5)
N3—Cu1—N2—C11	−164.9 (5)	C6—C1—C8—N1	−178.0 (3)
N1—Cu1—N2—C12	−101.1 (3)	C8—N1—C9—C10	−150.3 (5)
N3—Cu1—N2—C12	68.4 (3)	C9A—N1—C9—C10	−44.3 (8)
N1—Cu1—N2—C11A	41.7 (6)	Cu1—N1—C9—C10	46.0 (7)
N3—Cu1—N2—C11A	−148.9 (6)	N1—C9—C10—C11	−68.3 (9)
O1—Cu1—N3—C13	123.7 (6)	C12—N2—C11—C10	81.1 (7)
N1—Cu1—N3—C13	36.7 (10)	C11A—N2—C11—C10	−97 (2)
N2—Cu1—N3—C13	−60.0 (6)	Cu1—N2—C11—C10	−48.3 (8)
Cu1—O1—C2—C1	−6.7 (4)	C9—C10—C11—N2	69.8 (9)
Cu1—O1—C2—C3	174.05 (19)	C8—N1—C9A—C10A	132.2 (9)
C6—C1—C2—O1	−180.0 (2)	C9—N1—C9A—C10A	41.3 (8)
C8—C1—C2—O1	−2.5 (4)	Cu1—N1—C9A—C10A	−68.8 (12)
C6—C1—C2—C3	−0.8 (4)	N1—C9A—C10A—C11A	60.8 (18)
C8—C1—C2—C3	176.7 (3)	C11—N2—C11A—C10A	78 (2)
O1—C2—C3—C4	−179.9 (2)	C12—N2—C11A—C10A	75.1 (16)
C1—C2—C3—C4	0.9 (4)	Cu1—N2—C11A—C10A	−60.6 (15)
C7—O2—C4—C3	−1.2 (4)	C9A—C10A—C11A—N2	7(2)

Symmetry codes: (i) −*x*+1, −*y*, −*z*.

### Hydrogen-bond geometry (Å, °)

**Table:**

*D*—H···*A*	*D*—H	H···*A*	*D*···*A*	*D*—H···*A*
N2—H2A···O1^i^	0.91	2.14	2.999 (3)	157

Symmetry codes: (i) −*x*+1, −*y*, −*z*.
